# Miscellaneous

**Published:** 1890-04

**Authors:** 


### MISCELLANEOUS.

### TO DRIVE OUT FLIES.

I haven't a mosquito bar nor a screen door about my house, says Herbert A. Finley, in the St. Louis *Globe Democrat* and yet there are seldom any flies, and never any mosquitoes about it. I learned the secret of successful warfare against these pests when living in the swamps of Louisiana, where, summer or winter, mosquitoes swarm. For some years life was unendurable, and no meal could be eaten in peace. But all at once there was a change for the better. Bars and screens were often out of place, but there was almost an immunity from insects. I was batching at the time, and had just changed my colored boy. The new comer explained to me how he kept the "critters" away. He burnt small pieces of gum camphor on the cook stove, and used a secret preparation he called "sudekillo." When I got married and came to Missouri I imparted the secret to my wife, and as there is no patent on it that I know of, I would advise all fellow sufferers to go and do likewise. The gum camphor alone is ample for the purpose, and need only be used two or three times a day.

### A CAT RESCUES A BIRD.

A wonderful cat story which has the peculiar merit of being true is the following: A woman up town has a large number of birds which she keeps in an aviary built on the rear wall of the house. She also has a large Maltese cat who has proved herself so trustworthy and so kindly disposed toward the feathered pets that she had had for some time free range of the aviary whether the birds were in their separate cages or not.

A few days ago, while all the birds save one were shut in their cages, the owner heard a wild outcry in his aviary. Rushing to the door, she met her trusted cat coming out with this bird in her mouth. On the instant the next room was reached, however, the cat released her hold and the bird flew away frightened but wholly unharmed. On investigating the cause of the sudden confusion the woman found in the aviary a stray cat that had slipped in through a lowered sash. The house cat, seeing the danger, had snatched the bird in her mouth, so carefully as not to harm a feather, and carried it out of reach of the intruder.—*New York Sun.*

### A LITTLE MISUNDERSTANDING.

A gentleman from Indiana tells a story about Bayless W. Hanna, now United States minister to the Argentine Republic. Some months ago at Buenos Ayres a rich Spanish banker gave a dinner to some friends, and Mr. Hanna was seated on the right of the hostess. She inquired as to the health of Mrs. Hanna, who was not present, and asked how many children they had. Bayless, not understanding Spanish very well, thought she wanted to know the age of Mrs. Hanna, and said: "Forty-eight, madame." To his surprise the lady threw up her hands and exclaimed, "Gracios a Dios, que no tengo esposo Americano !" which, being translanted, is: "Thank God, I have not an American husband !" The next day the Spanish banker called on Mr. Hanna and said: "You astonished my wife yesterday when you told her you had forty-eight children." "Why, my dear sir," replied the minister, "I thought your wife inquired as to the age of Mrs. Hanna, and I gave it to her. I have only four children." The banker gave his wife the benefit of Judge Hanna's statement, but the story got out, and the judge has to stand up and take the jokes of his friends.

### THE MOTHER OF WASHINGTON.

Marion Harland, the well-known writer and journalist, has entered vigorously upon the praiseworthy undertaking of repairing and completing, according to the original plan, the unfinished monument designating the resting place of Mary, the mother of Washington, the base of which in a decayed and neglected state, occupies a place immediately adjacent to Fredericksburg, Va., where her remains were laid away more than a hundred years ago. It is now nearly half a century since the corner stone of the pedestal was laid with imposing ceremonies, in which Andrew Jackson, the then President, bore a conspicuous part.

It is never too late to do justice, and in the name of justice let the good work proceed to a finish which shall reflect honor alike upon the living and the illustrious dead.

Books for the registry of contributions have been opened at the office of *The Home Maker*, New York City.

### A CHURCH OF RIGHTEOUSNESS.

In the dark ages the clergy could do what they liked, and the laity would do what they were bid. But times have changed. Now with the progress of education, now when the results of science and literary research are brought within the reach of the masses, a Church has no chance of living unless it appeals to common sense, to reason, to the moral instincts of mankind. And just in proportion as it makes this appeal will it be strong and flourish and grow. Righteousness is essential—of all things most essential—to the welfare of men. They can get on well enough without any particular creed, they can get on well enough without special ritual; but without righteousness they perish! The Church, therefore, which insists most upon righteousness and less upon other things is the best Church. The Church which insists solely upon righteousness is the only Church that will not pass away.—Ex.

CHRISTIANITY AND THE W. C. T. U. — "MILWAUKEE, January 27. — The Woman's Christian Temperance Union here to-day received formal notice from the directors of the Young Men's Christian Association that the union could no longer occupy quarters in the Young Men's Christian Association building. The notice says that, having allied themselves with the Prohibition party, the women must be treated like any other political organization, and are therefore debarred from occupying quarters in the Young Men's Christian Association building."

